# Use of Dicyclopentadiene and Methyl Dicyclopentadiene for the Synthesis of Unsaturated Polyester Resins

**DOI:** 10.3390/polym13183135

**Published:** 2021-09-16

**Authors:** Alexandre Perrot, Jan Hyršl, Jan Bandžuch, Simona Waňousová, Jiří Hájek, Jan Jenčík, Tomáš Herink

**Affiliations:** 1 SYNPO a.s., S. K. Neumanna 1316, 532 07 Pardubice, Czech Republic; jan.bandzuch@synpo.cz; 2 ORLEN Unipetrol RPA s.r.o., Záluží 1, 436 70 Litvínov, Czech Republic; simona.wanousova@orlenunipetrol.cz (S.W.); tomas.herink@orlenunipetrol.cz (T.H.); 3 ORLEN UniCRE a.s., Záluží 1, 436 70 Litvínov, Czech Republic; jiri.hajek@orlenunicre.cz (J.H.); jan.jencik@orlenunicre.cz (J.J.); 4 Department of Chemistry, Faculty of Agrobiology, Food and Natural Resources, Czech University of Life Sciences Prague (CZU), Kamýcká 129, 165 00 Prague, Czech Republic

**Keywords:** dicyclopentadiene, methyl dicyclopentadiene, unsaturated polyester, water process

## Abstract

Dicyclopentadiene (DCPD) modified unsaturated polyester resins (UPRs) are mostly used for hulls, decks, and bathroom facilities. Main advantages of these polymers over orthophthalic or isophthalic polyesters are their relatively low shrinking, reduced styrene emission, lower cost, and fast curing in thin layers. On the other hand, once cured, these materials are more brittle and have lower glass transition temperatures and lower chemical resistance due to their different chemical constitutions. DCPD UPRs with standard grades are usually produced with high-quality DCPD (over a 85% purity) using the so-called “water process”, a synthesis consisting of two reaction steps. An adduct of maleic anhydride with DCPD is firstly formed with water, and then, it reacts with the other esterification monomers such as acids and glycols. DCPD raw materials used in this study were prepared by a unique distillation process developed by ORLEN Unipetrol and University of Chemistry and Technology, Prague. This technology allows producing a wide spectrum of DCPD quality by adjusting the content of another norbornene dimer: methyl dicyclopentadiene (MeDCPD). The influence of MeDCPD on unsaturated polyester properties was examined throughout this study. It has been discovered that in low concentrations, MeDCPD had a slight influence on flexural mechanical properties whereas its concentrations up to 65% led to a softer and brittle material. Nevertheless, by adjusting the unsaturation degree, it has been shown that MeDCPD may be successfully implanted in UPR formulation.

## 1. Introduction

Recently, low carbon footprint is becoming one of the most important subjects touching all technologies. As an output of this approach, lowering energy consumption based on fossil fuels is a crucial criterion in many industrial sectors. Therefore, material weight and energy consumption are more and more important not only for transportation, but also for general industry. Thermosetting composites with their unique properties and low energy needed for production become the most used material for metals and concretes replacement. In comparison with other curable resins, for instance epoxy, phenolic, melamine-formaldehyde, polyurethane, and polyimide used in composite structures, unsaturated polyesters (UPs) offer an easier possibility to be self-healed and recycled by using strong transesterification catalysts in the final cured resins [1,2,3]. Unsaturated polyester resins (UPRs) are a solution of linear or weakly branched UPs in reactive solvents, predominantly styrene [4]. UPRs are an important class of resins for thermosets. Indeed, the annual production in 2020 was about 5.2 million tons for approximately $8.4 billion.

UPR formulation can be effectively adapted for using pyrolysis products from polymeric waste or until now not usable by-products from fuel/crude oil distillations, and thus, crude oil products are used not only for fuel, but for the creation of desirable materials for technical purposes [4,5]. A well-known modification of UPR formulation is the dicyclopentadiene (DCPD) one. This norbornene monomer acts as a terminating agent against polyester chain growth. Unsaturated polycondensates modified by DCPD are generally more brittle with lower glass transition temperatures and lower chemical resistance [6,7,8]. On the other hand, these resins are less costly, having a lower shrinkage and viscosity [9,10]. In addition, these materials offer lower styrene emissions compared to standards UPRs. DCPD resins are mainly used in “low demanding applications”, for instance, hulls and decks or for domestic facilities typically bath tubes. Different synthesis routes are used for producing modified UPs, but the Diels–Alder method and the “water process” are the most common ones [11,12]. The former methods has been mostly used in the past 30 years, while the latter is nowadays more chosen due to its higher effectivity and lower costs [11,13,14]. Moreover, the so-called “water process” can be optimized to reduce gel particle content in the final product which is directly influenced by DCPD composition [15].

Monomeric DCPD quality varies from the production process. An innovative route has been developed by ORLEN Unipetrol in partnership with the University of Chemistry and Technology, Prague. The developed technology assumes the processing of light pyrolysis gasoline in a series of four distillation columns with a cascade of dimerization reactors. In the so-called Ethylene plant, light pyrolysis gasoline (LPyGas) corresponds to the bottom product of a debutanizer, where C4 hydrocarbons are separated from the mixture of hydrocarbons [16]. The LPyGas is a mixture of more than 130 components, mainly C5 and C6 hydrocarbons with high contents of benzene and toluene [17]. Cyclopentadiene (CPD) is dimerized in the debutanizer boiler and subsequently in reactors. As a result of diene dimerization, pyrolysis gasoline also contains dimers of CPD and methyl dicyclopentadiene (MeDCPD) and the corresponding codimers of these cyclic dienes with linear dienes (isoprene and piperylene).

**Figure 1:**
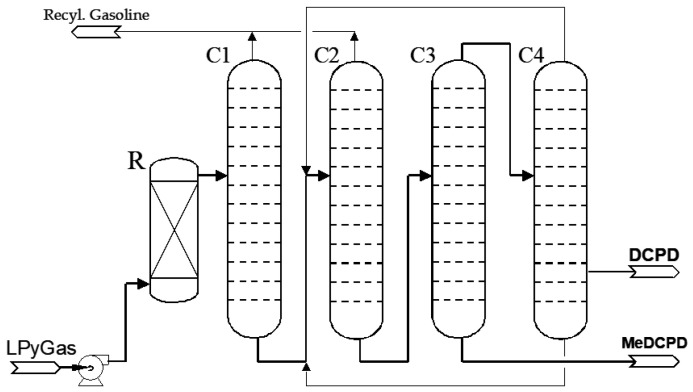
Process scheme for dicyclopentadiene (DCPD) recovery from light pyrolysis gasoline (LPyGas).

The process (see Figure 1) is flexibly designed to produce either 80% or 93–95% DCPD in campaigns. In the case of the isolation of 80–85% DCPD, the main impurities are the isomers of MeDCPD. In reality, it is possible to produce up to 26 kt of DCPD with a concentration of 80% and approximately 20 kt of DCPD with a concentration of 94% per year.

LPyGas is fed into a dimerizer (R) to convert remaining CPD to DCPD. The effluent from the dimerizer is fractionated in the first two columns (C1 and C2), where the C5–C9 hydrocarbons are separated from C10 hydrocarbons and heavier fractions. The bottom stream in the second column (C2), which contains 50–70% DCPD, is further distilled to yield DCPD as the overhead product of the third column (C3). Finally, the DCPD concentrate is fed to the fourth column (C4) to reach a quality related to color, trimer, peroxides content, etc. All streams which are not processed in DCPD are fed back to the Ethylene plant.

The technology induces the presence of MeDCPD in the final DCPD composition according to the desired quality but also in the distillation by-product in a concentration of approximately 55–65% (see. Figure 1). In comparison with other dimers observed in DCPD mixtures [18], MeDCPD could have an interesting added value in UPRs. However, this raw material has so far not been extensively studied in comparison to the plethora of documentation relating to DCPD. Its implementation in UP formulations is not well-known, although it is a major application for DCPD feedstock. A positive effect of MeDCPD in UP formulation would offers two main openings: the increase of polyester-grade DCPD quality and the reuse of the MeDCPD distillation by-product.

In this study, we studied the influence of MeDCPD in a standard UPR formulation. Neat resins properties were assessed for different compositions with an increasing amount of MeDCPD. Two kinds of DCPD composition have been tested: higher quality monomers up to 90% to evaluate the influence of MeDCPD in commonly used raw materials for UPRs and a technical by-product containing approximately 65% MeDCPD to determine the potential reuse of this substance.

## 2. Method Section

### 2.1. Materials

The raw materials used for the UP synthesis are listed in Table 1. In addition, the compositions of DCPD and MeDCPD are detailed in Table 2. The latter compositions were assessed by gas chromatography (GC) at ORLEN UniCRE (Litvínov-Záluží, Czech Republic).

Four UPs were synthesized from the above-described raw materials. These four resins were dissolved in styrene up to a 65% ± 1.5% dry matter. The characteristics of these resins are presented in Table 3.

Samples UP2, UPD90, UPD85 and UPMD65 had the same formulation basis to assess the influence of MeDCPD on polyester properties. The DPMD65 recipe was a model provided to exhibit the potential MeDCPD in a fully unsaturated UP structure. All resins were synthesized by a fusion process, i.e., without an azeotropic solvent, in a 1 L glass flask equipped with an anchor stirrer, a tempered filled column, a nitrogen inlet and a condenser. DCPD or MeDCPD unsaturated resins were prepared in a two-step route, which is often called “water process”, due to the anhydride ring opening with water in the first stage of synthesis. A constant stirring (150 rpm) was maintained during the whole process. DCPD or MeDCPD, tributyl phosphite, maleic anhydride and deionized water were firstly charged in a reactor, then heated to 120 °C and left for 30 min to reach an acid value of approximately 250 mg KOH/g. The aim of that first stage was to create a carboxy functional DCPD maleate adduct. In the second stage, the polycondensation took place: all remaining compounds such as phthalic anhydride, glycols as well as hydroquinone were fed and heated to the esterification temperature, i.e., 190 °C. The final carboxyl conversion was set at an acid value of 42 ± 5 mg KOH/g. Finally, the reaction mixture was cooled to 120 °C and rapidly prediluted in styrene. A completed dilution to a 65 wt. % dry matter was performed at room temperature after a sufficient homogenization. A standard UP polymer was prepared in the same way as a DCPD-modified material without the first synthesis stage.

Neat resins plates were produced to perform mechanical and physical tests. All samples were cured at 23 ± 2 °C for 24 h and 2 h at 120 ± 2 °C. The catalytic systems used for crosslinking is described in Table 4. The mentioned amounts corresponded to those of the diluted polyester.

### 2.2. Analysis

In this study, the influence of MeDCPD was evaluated in uncured and crosslinked samples. Concerning unreacted resins, color value, dynamic viscosity, dry matter, hydroxyl value, acid value, densities, average molecular weights, and reactivities were determined according to Table 5.

The viscoelastic, tensile and flexural properties, and densities of the cured materials were examined as well. Dynamic mechanical analysis (DMA) was performed on TA Instrument DHR-2 (New Castle, DE, USA), and the corresponding conditions are detailed in Table 6.

Both tensile and flexural properties were tested at 23 °C and 50% RH (relative humidity) from neat resin specimens on ZWICK/ROELLZ050-ZwickRoell, Ulm, Germany (except for UPMD65 specimens in bending, which were evaluated on Adamel-Lhomargy DY 36 (Testing machine Inc. New Castle, DE, USA). Experimental conditions are presented in Table 7. Finally, the cured densities were measured according to ČSN ISO 11831-1A. The volumetric shrinkage was evaluated from the uncured and cured material densities according to the following equation:

Volumetric shrinkage = 100 × (ρ_cured_ − ρ_uncured_)/ρ_uncured_.

## 3. Results and Discussion

### 3.1. Appearance

**Figure 2:**
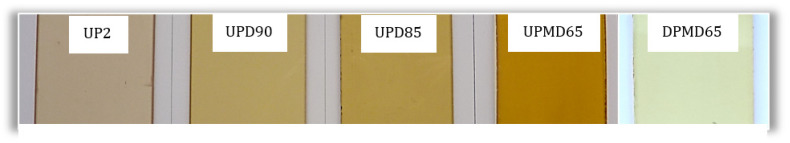
UPs appearances after curing.

The use of DCPD in the formulation of UPs resulted in color enhancements before and after curing (see Table 8 and Figure 2). This increase was more pronounced for UPMD65 and DPMD65 samples, which may be due to the higher content of heavier dyes in the MeDCPD65 monomer. The UPMD65 sample had an exceptionally higher color value caused by a more frequent sampling during synthesis. After curing, the DPMD65 material showed a slightly more yellowish color comparable to UPD90. Indeed, MeDCPD65 was a by-product of the DCPD distillation; therefore, it was not subject to further dyes purification. Since these substances were responsible for yellowing when the resin (or the monomer itself) was heated, a more yellowish color was expected for MeDCPD65-modified materials after each thermal exposure.

### 3.2. Uncured UPs Properties

**Figure 3:**
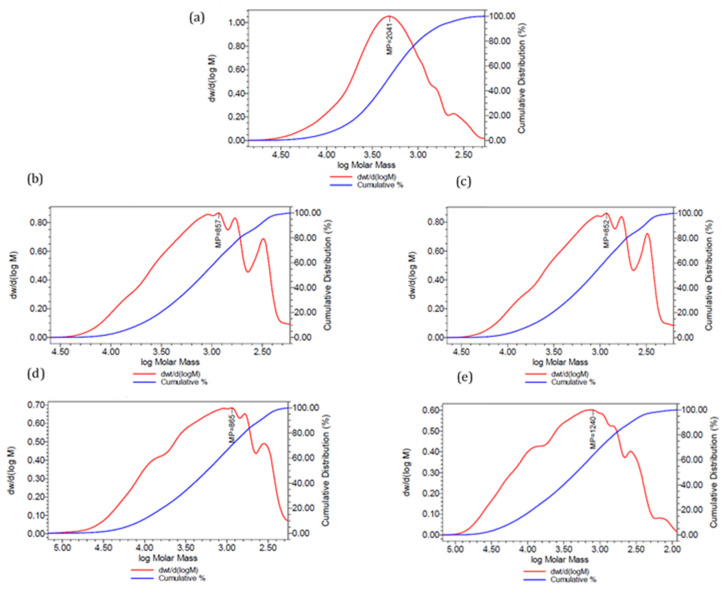
Differential dw/d(logM) and cumulative molar mass distributions of UP2 (**a**), UPD90 (**b**), UPD85 (**c**), UPMD65 (**d**), and DPMD65 (**e**).

In the first step, the characteristics of the liquid UPRs were examined. Table 9 represents all the basics properties of the synthesized resins. Acid values were divided into two series E1 and E2. The designation E1 corresponded to the acid value of the adduct DPCD (or MeDCPD)—maleate; E2 corresponded to the final polyester acid value. According to acid values, the conversion of carboxyl groups was similar for all samples. UPD polyesters and UPMD65 had a lower hydroxyl number probably due to the formation of DCPD–alcohol adducts and the preparation procedure. Morphology and chain length, i.e., molecular weights, were directly linked to acid and hydroxyl values. According to the GPC analysis, it was possible to classify formulation into three categories: standard UP, DCPD, and MeDCPD ones. Lower molecular weights were expected for UPD resins, because DCPD can act as a chain stopper. Moreover, in the UPMD65 (and UPD) formulation, a part of the reacted hydroxyl group content was replaced by DCPD or MeDCPD, which were also supposed to react with the carboxyl groups. Hence, if DCPD would not act as a chain stopper, a potential reduction of hydroxyl or pseudo hydroxyl reactant, may have led to a synthesis of longer chains inducing a higher weight average molecular weight (M_w_) than UP2. All samples had a similar dry matter; therefore, viscosity was directly influenced by M_w_. Figure 3 presents the molar mass distributions of the studied UPRs. As can be observed, DCPD-and MeDCPD-modified polyesters molecular weights distributions were shifted towards lower values, indicating a higher proportion of shorter chains. In addition, these polyesters contained a significant quantity of oligomers and showed a much broader distribution than UP2.

Finally, UPMD65 indicated the presence of macromolecules larger than 10 Daltons which was higher than UP, UPD, and UPMD samples [4,5]. This tendency towards the formation of longer chains might indicate that MeDCPD had a lower termination effect than DCPD. Another scenario would consist in the possible formation of polymaleate/fumarate chains due to the high content of maleate double bonds. This characteristic was even more pronounced for DPMD65, which contained only maleic anhydride and therefore a more reactive anhydride than the phthalic one.

As expected, volumetric shrinking was lower for DCPD samples, which is one of the main advantages of these monomers in UPRs. The same statement was observed for UPMD65 and DPMD65. A higher volumetric shrinking was observed for the latter formulation due to a higher unsaturation degree. This value was still 2% lower than the standard UP2, a non-fully UP, which was an advantage.

### 3.3. Reactivity

UPR reactivity is measured by monitoring the temperature development of a sample. Several key values are generally collected from this test: maximal exothermic temperature, “a time” corresponding to the time-lapse when the sample temperature is between 25 °C and 35 °C, “b time” corresponding to the time-lapse when the sample temperature is between 25 °C and the maximal exothermic temperature. The ratio between “b time” and “a time” was also mentioned. Table 10 shows the above-described parameters. As can be seen, DCPD monomers significantly decreased the maximal exothermic temperatures compared to UP2, which is an advantage for thicker material layers. Indeed, 20% and 25% decreases of exotherm were observed for UPD90 and UPD85 formulation, respectively. Overall, an increase of MeDCPD in DCPD monomers was followed by a drop of the maximal exothermic temperature. The DPMD65 sample had a higher maximal exothermic temperature due to a higher unsaturation degree.

**Figure 4:**
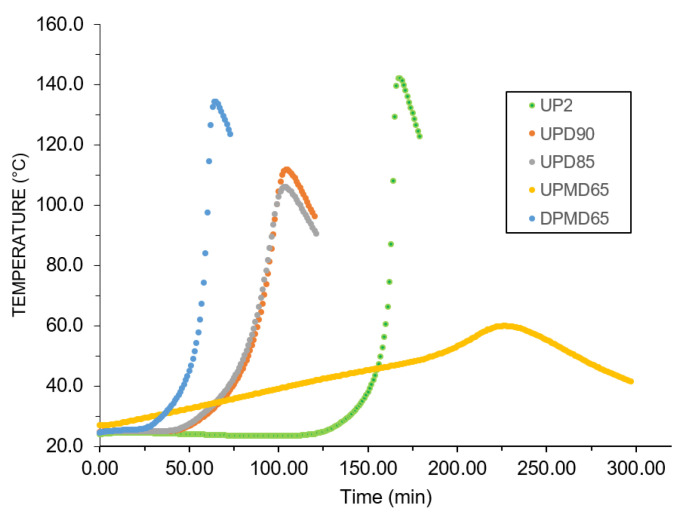
Temperature build-ups of the UPRs.

Shorter “a” and “b” times were observed for UPD and DPMD65 samples. This faster reactivity might be explained by the degradation of hydroquinone during synthesis and by the reactivity of the second double bond on DCPD during crosslinking. Figure 4 describes the temperature evolution as a function of time. As can be expected according to Table 10, the temperature build-up was faster for UPD samples. UPMD65 had a 30% higher “b time” value and a more than two times lower exotherm. This lower reactivity may be explained by the steric hindrance of the second double bond of MeDCPD induced by methyl groups. Indeed, saturated polymer chains due to the MeDCPD end chain might have given less advantageous copolymerization parameters than those associated with standard or DCPD-modified polyesters. Finally, the DPMD65 sample showed a higher exotherm as well a higher reactivity due to a higher unsaturation degree.

### 3.4. Dynamic Mechanical Analysis (DMA)

The viscoelastic Behaviors of UPs were evaluated by DMA (torsion measurement). Table 11 shows the glass transition temperatures of studied UPRs according to onset G’, maximal G”, and maximal tanδ. As can be noticed, a drop of 10 °C was observed for onset G” and maximal tanδ for UPD samples compared to for standard UP2. This result was expected according to the role of DCPD in polyester formulation. Moreover, DCPD modification promotes the cis configuration of the maleic double bound, which may be unfavourable for styrene copolymerization [19].

**Figure 5:**
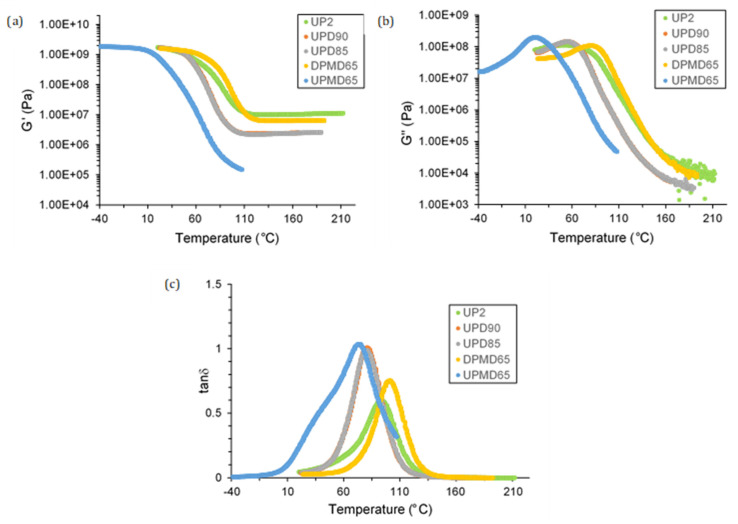
(**a**) Storage modulus; (**b**) loss modulus; (**c**) tanδ of UPRs.

Figure 5 represents the storage modulus, the loss modulus and tanδ values of the examined UPRs. As may be observed, the overall UP2 rubbery such plateau was higher than DCPD and MeDCPD polyester ones. This is followed by a less pronounced inflexion point with, for instance, two orders of magnitude in comparison to for UPMD65. The reduction of the network density was expected for DCPD-modified resins. According to the theory of rubber elasticity, the molecular weight between crosslinks is related to G’, polymer density, temperature, and universal gas content. Hence, storage modulus values at rubbery plateau gives a fair estimation of crosslink density. UPD90 and UPD85 showed a very similar viscoelastic Behavior. This trend should be confirmed by flexural and tensile properties.

An increase of MeDCPD amount in UPD90, UPD85, and UPMD65 formulations induced a decrease of T_g_ and an increase of tanδ peaks which are corresponding to the sol/gel ratio in a viscoelastic material. Therefore, a higher content of MeDCPD in a UPR could have led to a lower network density. Several hypotheses could be proposed to explain this phenomenon: the cured UPMD65 material might be a mixture of a highly crosslinked network plasticized with an unbounded linear polyester and polystyrene oligomers chains or a weekly crosslinked network with a lesser amount of uncross linked chains. A complex study of polymer morphology and copolymerization efficiency of MeDCPD should be provided to elucidate this issue.

DPMD65 showed a 100 times higher rubbery plateau than UPMD65, indicating a denser cross linkage. In this case, the potential lower reactivity of MeDCPD with styrene might be compensated by the full unsaturation of polyester backbone.

### 3.5. Tensile and Flexural Properties

Both tensile and flexural properties were evaluated. Table 12 and Table 13 summarize the samples flexural and tensile properties, respectively.

All the tested formulation showed the flexural strengths higher than the tensile strengths. That difference could be explained by material defects and geometry. Material defects were less involved in bending, since one half of the specimen was exposed to compression and fragile materials often show a higher resistance in bending than in tension. Sample thickness is also a key parameter, as it is squared in flexural strength calculations.

**Figure 6:**
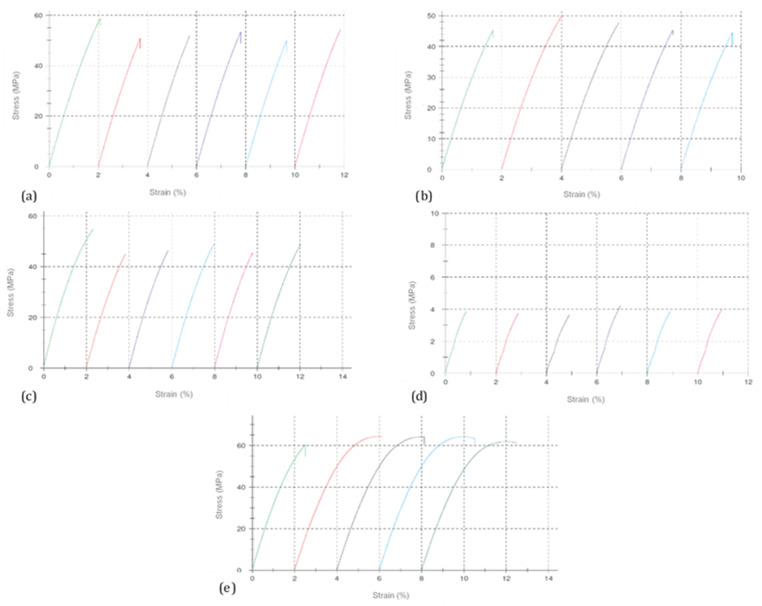
Tensile stress–strain curves of UPD90 (**a**), UPD85 (**b**), DPMD65 (**c**), UPMD65 (**d**), and UP2 (**e**).

All DCPD/MeDCPD-modified unsaturated resins showed a fragile rupture mode and a low elongation at break in comparison with standard UP2 (see Figure 6), a well-known influence of DCPD modification. Indeed, UPD90, UPD85, and DPMD65 showed a very similar rigidity, strength and elongation at break in both tension and bending. The UPD85 flexural strength was approximately 10 MPa lower than UPD polyesters and DPMD65, taking into account its standard deviation. This variation may be caused by specimen defects and geometry. DCPD85 differed from DCPD90 with a 5% higher MeDCPD content. Since both synthesized UP resins from those batches were similar, it could be assumed that at 23 °C, a DCPD85-modified polyester exhibited comparable mechanical properties with the DCPD90 counterpart.

A larger content of MeDCPD up to 65% (UPMD65) led to dramatic losses of rigidity and strength with almost a 10 times lower module for both tests, a 10 times lower strength in tension and almost a 20 times lower strength in bending. Strains at break for UPMD65 were very low for both types of load, which might rather indicate the presence of a weekly crosslinked network than a plasticized material. This drop was compensated by a higher unsaturation degree in the DPMD65 sample, which had a comparable strength and modulus in tension and bending with standard UP2. The DCPD-modified polyesters showed a higher standard deviation for flexural strength values, indicating a higher material heterogeneity.

## 4. Conclusions

DCPD-modified UPRs are well-known for their specific characteristics. Compared to unmodified UPs, these materials are brittle but show a lower shrinking, exotherm, and lower styrene emissions. MeDCPD obtained during the distillation process of DCPD have not been extensively studied so far, especially its influence on UPRs compared to standard UPRs or DCPD resins. In this work, we discovered that an increased amount of MeDCPD had an influence on different material stages. Regarding polymer morphology, higher molecular weights, and polydispersity have been found for MeDCPD-modified UPMD samples compared to its DCPD equivalents, i.e., UPD90 or UPD85. In addition, a lower reactivity, a lower rigidity, and a drastically more pronounced brittleness were noticed. These characteristics might have been induced by a weekly crosslink network and a plasticizing effect of unbounded polyester chains. Nevertheless, after the confrontation of UPD90 and UPD85, a small amount of MeDCPD up to 10% did not show a particular disadvantage in UPR properties overall. In addition, a fully UP based on 65% MeDCPD have exhibited encouraging properties, which may be an interesting way for further use. However, how to use this by-product of DCPD distillation is yet not explored. Indeed, MeDCPD-modified resins could be used or blended for low-cost UP composites. This implementation would increase the efficiency of the DCPD distillation line and reduce the cost of final polyester resins. Another interesting prospect of technical MeDCPD would be the usage in alkyd resins, a polycondensation material which can be modified by DCPD. In order to fully verify the feasibility of polyesters modification with MeDCPD, long-term stability as well as weathering and chemical resistance should be tested.

## Figures and Tables

**Table 1:** Raw materials for unsaturated polyester (UP) synthesis and applications.

	Name	Acronym	Supplier
UP synthesis	Phthalic anhydride	PA	Spolchemie (Ústí nad Labem, Czech Republic)
	Maleic anhydride	MA	
	1,2-propylene glycol	PG	
	Diethylene glycol	DEG	
	Tributyl phosphite	TBF	
	90% dicyclopentadiene (DCPD)	DCPD90	ORLEN Unipetrol (Litvínov-Záluží, Czech Republic)
	85% DCPD	DCPD85	
	65% methyl dicyclopentadiene (MeDCPD)	MeDCPD65	
	Styrene	St	Spolchemie (Ústí nad Labem, Czech Republic)
Catalytic system	Hydroquinone	HQ	
	Methyl ethyl ketone peroxide	MEKP	
	1% cobalt octoate in toluene	Co	

**Table 2:** Dicyclopentadiene (DCPD) and methyl dicyclopentadiene (MeDCPD) compositions.

	DCPD90	DCPD85	MeDCPD65
CPD	0.0	0.0	0.0
Benzene	0.0	0.0	0.0
Toluene	0.0	0.0	0.0
Codimers A ^a^, sum	1.2	1.1	0.0
DCPD, sum	90.7	84.3	20.6
Codimer B ^b^	1.4	1.9	2.0
MeDCPD, sum	5.5	10.8	67.2
DiMeDCPD, sum	0.2	0.3	2.5

^a^ Codimers A is a group of isomers of CPD–piperylene and CPD–isoprene with a lower boiling point than DCPD. ^b^ Codimer B is the isomer of the CPD–isoprene with a higher boiling point than DCPD.

**Table 3:** Unsaturated polyester resins (UPRs) formulations.

Polyester Sample Name	Anhydrides	Glycols	Dicyclopentadiene Batch	Theoretical Parameter
UP2 (standard)	PA/MA ^a^	PG/DEG ^b^	/	N ^c^ = 1.20 R ^d^ = 1.10
UPD90			DCPD90	
UPD85			DCPD85	
UPMD65			MeDCPD65	
DPMD65	MA	DEG	MeDCPD65	R = 1.12

^a^ Phthalic anhydride (PA)/Maleic anhydride (MA). ^b^ propylene glycol (PG)/Diethylene glycol (DEG). ^c^ N is defined as the ratio between the phthalic anhydride equivalent and the maleic anhydride equivalent. ^d^ R is defined as the ratio between all hydroxy equivalents and all carboxy equivalents.

**Table 4:** Catalytic system for UP curing.

Compound	Amount
HQ	0.0016%
Co	1%
MEKP	1%

**Table 5:** Liquid resin (uncured) characterization.

Analysis	Standard/Method	Instrument	Conditions
Color value	Gardner	LICO 620 (Hach Company, Loveland, CO, USA)	23 °C
Dynamic viscosity	ISO 2284	Brookfield CAP2000+ (AMETEK Brookfield, Middleboro, MA, USA)	25 °C/100 rpm/C1 (cone-plate)
Dry matter	Internal method	Owen Binder (BINDER GmbH, Tuttlingen, Germany)	150 °C for 30 min (1 g of resin with 3 drops of a 1% hydroquinone solution in ethanol)
Density	ČSN EN ISO 1675	Non relevant	25 °C
Acid value	ČSN EN ISO 2114	Mettler Toledo T50 excellence titrator (Mettler Toledo, Colombus, OH, USA)	23 °C
Hydroxyl value	Internal method PP12	Mettler Toledo T50 excellence titrator (Mettler Toledo, Colombus, OH, USA)	23 °C
Gel permeation chromatography (GPC)	ČSN ISO 13885-1	Waters e2695 Refractive Index detector Waters 2414 (Waters corporation, Milford, MA, USA)	Column: PLgel 5µm MIXED-C 300 × 7.5 mm (Agilent technologies)
Reactivity (monitoring of the sample’s temperature build-up)	Internal method	MULTILOGGER M 1200E (COMET system, Rožnov pod Radhoštěm Czech Republic)	15 g polyester, 1% MEKP and 1% Co; 25 ± 2 °C

**Table 6:** Dynamic mechanical analysis (DMA) parameters.

Analysis Type	Temperature Ramp; Temperature Range	Frequency; Maximum Deformation	Specimen Dimensions
Torsion	3 °C/min; 25–180 °C	1 Hz; 1%	26 mm × 10 mm × 3 mm

**Table 7:** Tensile and flexural analysis conditions.

Analysis Type	Tension	Bending
Standard	ČSN EN ISO 527-2	ČSN EN ISO 178 Method B
Speed	Module: 1 mm/min Strength: 2 mm/min	Module: 2 mm/min Strength: 10 mm/min
Load cell	50 kN	50 kN
Fixture	Pneumatic Zwick 10 kN (ZwickRoell, Ulm, Germany)	3-point bending
Extensometer	MultiXtens (ZwickRoell, Ulm, Germany)	MultiXtens (ZwickRoell, Ulm, Germany)

**Table 8:** Color values of the uncured UPs (GARDNER).

UP2	UPD90	UP85	UPMD65	DPMD65
1.5	4.8	5.3	9.2	6.0

**Table 9:** Basic UPR properties.

	UP2	UPD90	UPD85	UPMD65	DPMD65
E1 acid value (mg KOH/g)	Non relevant ^a^	245	256	254	Not measurable ^b^
E2 acid value (mg KOH/g)	43	44	42	50	40
Hydroxyl value (mg KOH/g)	47	4	12	0	32
Mn (g/mol)	1400	800	800	1000	1000
Mw (g/mol)	3400	2300	2200	4000	5900
Polydispersity	2.4	2.9	2.8	4.0	5.9
Dry matter (%)	64.9	64.6	65.2	64.9	64.4
Dynamic viscosity (mPa·s)	402	274	274	615	651
Density (uncured resin)	1.134	1.115	1.116	1.117	1.120
Density (cured resin)	1.231	1.198	1.200	1182	1.204
Volumetric shrinking (%)	9	7	8	6	7

^a^ UP2 synthesis was a one step route. ^b^ At the end of step one^,^ DMPD65 sample mixture was not homogeneous, therefore acid value was not measurable.

**Table 10:** Reactivity parameters of UPRs.

	a Time (Min)	b Time (Min)	b Time/a Time Ratio	T Peak (°C)
UP2	146	168	1.2	142
UPD90	67	104	1.8	112
UPD85	65	103	1.6	106
UPMD65	69	226	3.3	60
DPMD65	55	79	1,4	127

**Table 11:** Glass transition temperatures of UPRs.

	T_g_ at Maximal tanδ (°C)	T_g_ at Onset G′ (°C)	T_g_ at Maximal G″ (°C)
UP2	93	66	95
UPD90	81	53	55
UPD85	80	53	55
UPMD65	73	21	16
DPMD65	101	78	82

**Table 12:** Tensile properties of the UPRs.

Sample	Young Modulus	Tensile Strenght	Strain at Break
	E (GPa)	σ (MPa)	ε_max_ (%)
UP2	3.14 ± 0.13	63.0 ± 1.8	3.9 ± 0.8
UPD90	3.16 ± 0.11	48.3 ± 3.6	2.0 ± 0.2
UPD85	3.16 ± 0.05	46.6 ± 2.2	1.8 ± 0.1
UPMD65	0.46 ± 0.03	3.9 ± 0.2	0.9 ± 0.05
DPMD65	3.46 ± 0.03	53.2 ± 3.2	3.2 ± 1.8

**Table 13:** Flexural properties of the UPRs.

Sample	Thickness	Flexural Modulus	Flexural Strength	Strain at Break
	d (mm)	E_f_ (GPa)	σ_f_ (MPa)	ε_max_ (%)
UP2	3.52 ± 0.1	3.10 ± 0.1	121.4 ± 1.1	4.8 ± 0.1
UPD90	3.59 ± 0.1	3.03 ± 0.1	115.6 ± 6.8	2.5 ± 0.6
UPD85	3.76 ± 0.04	3.13 ± 0.2	77.3 ± 19.0	1.1 ± 0.6
UPMD65	3.91 ± 0.04	0.42 ± 0.02	5.7 ± 0.4	1.0 ± 0.1
DPMD65	3.74 ± 0.01	3.62 ± 0.1	117.2 ± 17.6	2.3 ± 1.1

## Data Availability

The data presented in this study are available on request from the corresponding author. The data are not publicly available due to the preservation of ORLEN Unipetrol/ORLEN Unicre and Synpo know-how.

## Abbreviations

List of abbreviations

Co	1% solution of cobalt naphthenate in toluene
DCPD	dicyclopentadiene
DCPD90	90% dicyclopentadiene
DCPD85	85% dicyclopentadiene
DEG	diethylene glycol
DiMeDCPD	dimethyl dicyclopentadiene
DPMD65	model of fully unsaturated polyester resin modified with 65% MeDCPD
HQ	hydroquinone
LPyGas	light pyrolysis gas
MA	maleic anhydride
MeDCPD	methyl dicyclopentadiene
MeDCPD65	65% methyl dicyclopentadiene
MEKP	methyl ethyl ketone peroxide
M_n_	number averaged molecular weight
M_W_	weight average molecular weight
N	the ratio of the phthalic anhydride equivalent and the maleic anhydride equivalent
PA	phthalic anhydride
PG	1,2-propylene glycol
R	the ratio of the hydroxyl equivalent and the carboxyl equivalent
St	styrene
TBF	Tributyl phosphite
T_g_	glass transition temperature
UP	unsaturated polyester
UPR	unsaturated polyester resin
UP2	standard unsaturated polyester resin
UPMD65	modified UP2 standard unsaturated polyester resin with MeDCPD65
UPD85	modified UP2 standard unsaturated polyester resin with DCPD85
UPD90	modified UP2 standard unsaturated polyester resin with DCPD90
